# Quality assurance in specialized headache units in Spain: an observational prospective study

**DOI:** 10.1186/s10194-019-1020-1

**Published:** 2019-06-25

**Authors:** Patricia Pozo-Rosich, Alba Martínez-García, Julio Pascual, Emilio Ignacio, Ángel L. Guerrero-Peral, José Balseiro-Gómez, Jesús Porta-Etessam, Germán Latorre-González, Almudena Layos-Romero, César Lucas, José J. Mira

**Affiliations:** 1 Headache Unit, Neurology Department, Hospital Universitari Vall d’Hebron, Barcelona, Spain; 2 Headache Research Group, Vall d’Hebron Research Institute (VHIR), Universitat Autònoma de Barcelona, Barcelona, Spain; 3 Calitè Research Group, Miguel Hernández University, Elche, Spain; 4 Marqués de Valdecilla Hospital, Santander, Spain; 5 Valdecilla Biomedical Research Institute (IDIVAL), Santander, Spain; 6 Cádiz University, Cádiz, Spain; 7 Headache Unit, Neurology Department, University Hospital of Valladolid, Valladolid, Spain; 8 Institute for Biomedical Research of Salamanca (IBSAL), Salamanca, Spain; 9 Neurology Department, University Hospital of Getafe, Getafe, Spain; 10 Headache Unit, Neurology Department, San Carlos Hospital, Madrid, Spain; 11 Neurology Department, University Hospital of Fuenlabrada, Madrid, Spain; 12 Neurology Department, University Hospital of Albacete, Albacete, Spain; 13 Neurology Department, Virgen de la Arrixaca University Hospital, Murcia, Spain; 14 Alicante-Sant Joan Health District, Alicante, Spain; 15 Health Services Research in Chronic Illnesses Network, REDISSEC, Alicante, Spain

**Keywords:** Headache, Indicators, Quality assurance, Patient safety

## Abstract

**Objective:**

To assess the quality of the therapeutic approach in Specialized Headache Units in Spain.

**Methods:**

An observational (prospective) study was conducted. Anonymized data of 313 consecutive patients during a defined period of time were analyzed and a comparison of performance in 13 consensual quality indicators between Specialized Headache Units and neurology consultations was calculated. Specialized Units and neurology consultations represented the type of provision that Spaniards receive in hospitals.

**Results:**

The consensus benchmark standard was reached for 8/13 (61%) indicators. Specialized Headache Units performed better in the indicators, specifically in relation to accessibility, equity, safety, and patient satisfaction. Patients attended in Specialized Headache Units had more complex conditions.

**Conclusion:**

Although there is variability among Specialized Headache Units, the overall quality was generally better than in traditional neurology consultations in Spain.

## Introduction

Headache is a very common health problem that requires an effective and coordinated response from healthcare provision, both from Primary Care and by Specialized Headache Units in hospitals.

Diagnostic criteria and recommended treatment guidelines have been established [1, 2], although in clinical daily practice there seems to be great differences in how the response to headache is organized by the different health systems. Most regional health authorities do not have established protocols for the treatment of headaches, which affects, particularly, the variability of the quality of health care of these patients. However, the adequate treatment of headache is of interest to everyone because of its overall impact on people’s health and well-being, the economic consequences that inadequate treatment or care might have, and also because of the social and work implications that this ailment entails.

On March 2004, the Global Campaign against Headache [3] was launched, spearheaded by the Lifting the Burden (LtB) organization along with the World Health Organization, which laid the foundations for international collaboration in order to reduce the fragmentation of organizational, diagnostic and therapeutic approaches throughout the world and, particularly, in Europe [4]. Within this cooperation framework, with the participation of the European Headache Federation (EHF) a study was carried out to identify the dimensions in the quality of care for headache patients that ought to be taken into consideration. This study was carried out through a systematic review of the available evidence [5] and consensus methods [6, 7]. For each dimension, a proposal of quality indicators was established that, in total, comprised 30 indicators grouped into 9 dimensions, ranging from equipment and infrastructure to assessment of the cost of the care mechanism.

The proposal of LtB and EHF has been easily understood and has been considered useful for establishing comparisons between approaches to the care afforded to headache patients by professionals in different countries. So far, it has been applied in 14 centers in 11 European countries (including Turkey), developing five instruments according to this set of indicators for the collection of information [8]. This evaluative approach has also been used when comparing the approaches of two centers of attention to headache patients in Germany (89 patients) and Portugal (50 patients) [9] and for evaluating, in Italy [10], six units specialized in headache diagnosis and treatment (calculating the indicators based on the records of 360 patients). In most cases, the indicators used were related to the mechanism’s structure and to the care process.

The Eurolight study [11] has analyzed the organization and treatment of migraine patients in Europe. Other studies [12] have done sub-analysis using the data base of the Eurolight project. In this case, a cross-sectional study was carried out, although with a different sampling method depending on the country. The data allowed to compare the prevalence, use of medical services and prescriptions to 3466 migraine patients among 10 European countries. In particular, they compared the use of triptans in the symptomatic treatment of migraine by medical specialists, general practitioners and other non-medical therapists. The number of patients suffering migraine at least 5 days a month was also considered, in whom a preventive treatment had been indicated by these three professional groups.

A previous study [13], conducted in Spain, to reach a consensus on outcome indicators in the diagnostic and therapeutic approach for Specialized Headache Units, defined 13 indicators that are applicable to patients with primary chronic headaches, the most common ailment to be treated at specialist headache units in Spanish hospitals.

The objective of this study was to evaluate the quality of the therapeutic approach in Specialized Headache Units in Spain and compare their performance with traditional neurology consultations by using these agreed outcome indicators.

## Methods

An observational (prospective) study was conducted. Anonymized data of consecutive patients during a defined period of time were analyzed. This study was approved by the Research Ethic Committee named CEIM Valladolid Este.

### Headache specialist units

In Spain, traditionally most hospitals do not have specialized units in headache diagnosis and treatment. In recent years, the number of these units has increased, although their number is still scarce. In both public and private hospitals, it is more usual to have specialized consultations in the neurology services.

### Participants

Three specialized units in the diagnosis and treatment of patients with headache, integrated into the neurology services, and other four Spanish neurology services, agreed to participate. Table 1 describes the characteristics of them. The selection of specialized units and neurology consultations involved different hospitals’ size and resources and, represents the type of assistance that Spaniards receive in the hospitals.

**Table 1:** Characteristics of the Specialized Headache Units taking part in this study

	Fuenlabrada^1^	Getafe^2^	Albacete^3^	Hosp Virgen de la Arrixaca^4^	Vall d’Hebron^5^	San Carlos^6^	HCUV^7^
Number of surgeries (rooms for consultation)	1	1	1	1	1 + 1 part-time	3 + 1 part time	2
Number of patients attended in the unit per year	1000	510	600	950	3200	8000	2500
Number of neurologists	1	2	2 part-time	1	1 + 1 part-time	3 + 2 part time	2
Number of nurses	0	1	0	0	0	1	1
Specialized Headache Units	NO	NO	NO	NO	YES	YES	YES

^1^Fuenlabrada University Hospital, Fuenlabrada, Madrid

^2^Getafe University Hospital, Getafe, Madrid

^3^Albacete University Hospital Complex, Albacete

^4^Virgen de la Arrixaca University Hospital, Murcia

^5^Vall d’Hebron University Hospital, Barcelona

^6^San Carlos Clinical Hospital, Madrid

^7^Valladolid University Clinical Hospital, Valladolid

### Quality indicators definition

The 13 agreed indicators [13] were measured in order to assess the quality of the therapeutic indications. These indicators cover five aspects (effectiveness, patient-centered care, patient safety, accessibility and adequacy) and were developed based on a review of the literature on the proposal of quality indicators and the work of a Core Group, composed of eight specialists in Neurology. Table 2 shows the aspects, the definition of each indicator and the data sources that allow the measurement.

**Table 2:** Indicators used in this study

Definition	Indicator type	Dimension
CEF1. Percentage of patients diagnosed with primary chronic headache who, in a period of one year, attend the Emergency Room due to headache or complications of treatment.	Results	Effectiveness
CEF2. Percentage of patients with chronic migraine who improve in the assessment according to the MIDAS scale, pre-post measure, at three months following therapeutic intervention.	Results	Effectiveness
CEF3. Percentage of patients with primary chronic headache who progress to episodic headache (< 15 days of pain per month) after treatment.	Results	Effectiveness
CEF4. Percentage of patients who obtain more than 7/10 points on scales that measure satisfaction with the care provided.	Results	Patient Centered Care
CEF5. Percentage of patients with primary chronic headache treated in the Unit with at least one period of sick leave due to headache in the previous three months.	Results	Patient Centered Care
CEF6. Percentage of patients with primary headache receiving preventive treatment three months after their prescription.	Results	Patient Centered Care
CEF7. Percentage of patients with primary headache in analgesic treatment for more than 10 days a month in the last 3 months in the Specialized Headache Units.	Process	Patient Safety
CEF8. Percentage of patients with a diagnosis of primary headache in the Specialized Headache Units or Doctor’s Surgery, without repetition of neuroimaging studies in a one-year period.	Process	Patient Safety
CEF9. Percentage of patients with primary chronic headache in non-recommended symptomatic treatment.	Results	Patient Safety
CEF10. Percentage of patients with primary chronic headache who suffer one or more adverse events which resulted in treatment being withdrawn.	Results	Patient Safety
CEF11. Percentage of patients with cluster headache attended within seven days from the onset.	Process	Accessibility
CEF12. Percentage of pregnant patients with primary headache attended within 15 days of the positive pregnancy test.	Process	Accessibility
CEF13. Percentage of patients with cluster headache episode who have home oxygen therapy	Structure	Equity

### Quality indicators assessment

A three-item questionnaire was used to determine the ease with which data could be accessed to calculate the indicators, the reliability of such data and to assess the suitability of the indicators that were calculated using these data. The three following questions were used: “Easy access to the data to calculate the indicators”, “The data sources to calculate the indicators are reliable” and “The relevance of the indicators that have been calculated” (Appendix I).

### Data collection

Data collection was carried out during the months of April to June 2018. The information for the calculation of the indicators was taken from each patient’s clinical history. The information was encoded by the head of each unit in each center. Data were obtained from a minimum of 45 consecutive patients (both patients who attended the consultation for the first time, as well as patients who attended the follow-up appointment). A total of 315 cases were collected and their data extracted following this procedure. Sample was calculated considering 95% confidence level and 80% statistical power.

For each indicator, anonymized data were collected (number of patients that met the definition of the indicator) that allowed to quantify numerators and denominators of each of the indicators. Each Specialized Headache Units entered data into spreadsheets, which were then transferred to the Miguel Hernández University of Elche (data collection center) where the indicators were calculated.

### Data analysis

A descriptive analysis and comparison of data using t-test were performed in function of the complexity attended in each Specialized Headache Units. Indicators were compared with the quality standard agreed upon in a previous study [13]. No hypotheses were statistically tested.

## Results

Anonymized data were encoded from a total of 313 patients. In one hospital only 43 valid cases were encoded, as in two cases data were not complete and were discarded.

The greatest variability in the indicators analyzed (Table 3) was observed in the percentage of patients diagnosed with primary chronic headache attending the Emergency Room (CEF1), with primary headache without repetition of neuroimaging studies (CEF8), with non-recommended symptomatic treatment (CEF9), pregnant women attended within 15 days of the positive pregnancy test (CEF12) and patients with active cluster headaches that have home oxygen therapy (CEF13).

**Table 3:** Results in the indicators analyzed in each of the participating Specialized Headache Units

Indicators studied	Global#	Fuenlabrada^1^	Getafe^2^	Albacete^3^	Murcia^4^	Vall d’Hebron^5^	San Carlos^6^	HCUV^7^	CV (%)
CEF1. Percentage of patients diagnosed with primary chronic headache who, in a period of one year, attend the Emergency Room due to headache or complications of treatment.	28.7	11.1	28.9	46.7	67.4	15.6	17.8	15.6	72
CEF2. Percentage of patients with chronic migraine who improve in the assessment according to the MIDAS scale, pre-post measure, at three months following therapeutic intervention.	67.8*	86.7	64.4	66.7	67.4	53.7^+^	80	57.8	15
CEF3. Percentage of patients with primary chronic headache who progress to episodic headache (< 15 days of pain per month) after treatment.	67.4	77.8	60.0	64.4	83.7	66.7	66.7	53.3	15
CEF4. Percentage of patients who obtain more than 7/10 points on scales that measure satisfaction with the care provided.	87.9	93.3	80.0	80.0	83.7	88.9	97.8	91.1	8
CEF5. Percentage of patients with primary chronic headache treated in the Unit with at least one period of sick leave due to headache in the previous three months.	14.1*	11.1	8.9	8.9	25.6	11.1^+^	15.6	20	45
CEF6. Percentage of patients with primary headache receiving preventive treatment three months after their prescription.	84.7*	77.8	75.6	84.4	76.7	100^+^	86.7	93.3	8
CEF7. Percentage of patients with primary headache in analgesic treatment for more than 10 days a month in the last 3 months in the Specialized Headache Units.	39.6*	40	48.9	48.9	34.9	48.9^+^	24.4	42.2	23
CEF8. Percentage of patients with a diagnosis of primary headache in the Specialized Headache Units or Doctor’s Surgery, without repetition of neuroimaging studies in a one-year period.	14.4	46.7	20.0	4.4	18.6	0.0	4.4	6.7	112
CEF9. Percentage of patients with primary chronic headache in non-recommended symptomatic treatment.	20.2*	8.9	35.6	35.6	9.3	55.6	13.3	4.5	107
CEF10. Percentage of patients with primary chronic headache who suffer one or more adverse events which resulted in treatment being withdrawn.	30.3	46.7	37.8	35.6	25.6	44.4	13.3	8.9	49
CEF11. Percentage of patients with cluster headache attended within seven days from the onset.	50.6	73.7	68.9	2.2	50.0	100	78.9	58.8	61
CEF12. Percentage of pregnant patients with primary headache attended within 15 days of the positive pregnancy test.	24.6	4.5	31.1	20.0	97.7	100	66.7	77.8	155
CEF13. Percentage of patients with cluster headache episode who have home oxygen therapy.	45.0	93.3	11.1	8.9	25.0	66.7	78.9	76.5	75

CV coefficient of variation

# Average calculated considering the data set of all the hospitals

*Does not include data from the Vall d’Hebron University Hospital that applied a different timescale criterion

+ At 6 months

^1^Fuenlabrada University Hospital, Fuenlabrada, Madrid

^2^Getafe University Hospital, Getafe, Madrid

^3^Albacete University Hospital Complex, Albacete

^4^Virgen de la Arrixaca University Hospital, Murcia

^5^Vall d’Hebron University Hospital, Barcelona

^6^San Carlos Clinical Hospital, Madrid

^7^Valladolid University Clinical Hospital, Valladolid

Overall, in 8/13 (61%) indicators, the consensus benchmark standard was reached (Table 4). In five (38%) indicators, all the units met the pre-set quality benchmark, in two (15%) indicators, only three units of the seven participants reached the standard, in one indicator (8%), only one unit met the standard and in one indicator (8%). None of the units reached the standard (Table 4). The indicators assessment ranged between 8 to 9.5 (Appendix II).

**Table 4:** Level of compliance with standards for the indicators assessed

Indicators studied	Indicators that meet the benchmark standard (n/N)	Benchmark*	Overall compliance of the standard (YES/NO)
CEF1. Percentage of patients diagnosed with primary chronic headache who, in a period of one year, attend the Emergency Room due to headache or complications of treatment.	5/7	< 30%	YES
CEF2. Percentage of patients with chronic migraine who improve in the assessment according to the MIDAS scale, pre-post measure, at three months following therapeutic intervention.	7/7	> 50%	YES
CEF3. Percentage of patients with primary chronic headache who progress to episodic headache (< 15 days of pain per month) after treatment.	7/7	> 50%	YES
CEF4. Percentage of patients who obtain more than 7/10 points on scales that measure satisfaction with the care provided.	7/7	> 70%	YES
CEF5. Percentage of patients with primary chronic headache treated in the Unit with at least one period of sick leave due to headache in the previous three months.	7/7	< 30%	YES
CEF6. Percentage of patients with primary headache receiving preventive treatment three months after their prescription.	7/7	> 50%	YES
CEF7. Percentage of patients with primary headache in analgesic treatment for more than 10 days a month in the last 3 months in the Specialized Headache Units.	1/7	< 30%	NO
CEF8. Percentage of patients with a diagnosis of primary headache in the Specialized Headache Units or Doctor’s Surgery, without repetition of neuroimaging studies in a one-year period.	0/7	> 70%	NO
CEF9. Percentage of patients with primary chronic headache in non-recommended symptomatic treatment.	4/7	< 30%	YES
CEF10. Percentage of patients with primary chronic headache who suffer one or more adverse events which resulted in treatment being withdrawn.	5/7	< 40%	YES
CEF11. Percentage of patients with cluster headache attended within seven days from the onset.	3/7	> 70%	NO
CEF12. Percentage of pregnant patients with primary headache attended within 15 days of the positive pregnancy test.	3/7	> 70%	NO
CEF13. Percentage of patients with cluster headache episode who have home oxygen therapy.	4/7	> 50%	NO

*Source: Carrillo I, Pozo-Rosich P, Guilabert M, Ignacio E, Pascual J, Porta J, Guerrero A, Mira JJ. Portfolio of services and basic chart of quality indicators for the Specialized Headache Units. Consensus Study. Journal of Neurology. 2018;68:118-122.

Headaches Specialized Units showed better performance in the indicators than traditional headaches consultations. Statistically significant differences suggested that Headaches Specialized Units achieve a minor number of emergency visits (CEF1), repetition of neuroimaging procedures (CEF8), and adverse events (CEF10). In turn, they achieve major patient satisfaction (CEF4), number of patients involved in preventive treatment (CEF6), patient undergoing oxygen therapy at home (CEF13) and, showed better response capacity (CEF11, CEF12) (Table 5). These results represent that half of patients treated at Specialized Units need to go to the hospital emergency departments as consequence of treatment complications, comparing with traditional care; seven times less repeat neuroimage tests in one-year-period; and response capacity and benefits for specific conditions were better.

**Table 5:** Results in the indicators according to the complexity of cases attended in the centers participating in the study

Indicators in the study	Headaches Specialized Units (N patients = 135)		Headaches consultations (N patients = 178)			
	Mean	Standard Deviation	Mean	Standard Deviation	Confidence interval on the difference between means	*P*-Value
CEF1. Percentage of patients diagnosed with primary chronic headache who, in a period of one year, attend the Emergency Room due to headache or complications of treatment.	16.3	37.1	38.2	48.7	12.0, 31.8	0.0001
CEF2. Percentage of patients with chronic migraine who improve in the assessment according to the MIDAS scale, pre-post measure, at three months following therapeutic intervention.	62.7	48.5	71.3	45.3	−2.1, 19.3	0.113
CEF3. Percentage of patients with primary chronic headache who progress to episodic headache (< 15 days of pain per month) after treatment.	62.2	48.7	71.3	45.3	−1.4, 19.6	0.092
CEF4. Percentage of patients who obtain more than 7/10 points on scales that measure satisfaction with the care provided.	92.6	26.3	84.3	36.5	−15.6, − 1.0	0.020
CEF5. Percentage of patients with primary chronic headache treated in the Unit with at least one period of sick leave due to headache in the previous three months.	14.8	35.6	13.5	34.2	−15.6, −6.5	0.738
CEF6. Percentage of patients with primary headache receiving preventive treatment three months after their prescription.	92.6	26.3	78.6	41.1	−21.9, −6.0	0.0001
CEF7. Percentage of patients with primary headache in analgesic treatment for more than 10 days a month in the last 3 months in the Specialized Headache Units.	34.8	47.8	43.3	49.7	−2.5, 19.4	0.129
CEF8. Percentage of patients with a diagnosis of primary headache in the Specialized Headache Units or Doctor’s Surgery, without repetition of neuroimaging studies in a one-year period.	3.7	18.9	22.5	41.8	11.1, 26.4	0.0001
CEF9. Percentage of patients with primary chronic headache in non-recommended symptomatic treatment.	17.2	37.8	22.5	41.9	−3.7, 14.3	0.242
CEF10. Percentage of patients with primary chronic headache who suffer one or more adverse events which resulted in treatment being withdrawn.	22.2	41.7	36.5	48.3	4.1, 24.5	0.005
CEF11. Percentage of patients with cluster headache attended within seven days from the onset.	73.2	44.9	42.5	49.6	−48.1, −13.2	0.0001
CEF12. Percentage of pregnant patients with primary headache attended within 15 days of the positive pregnancy test.	76.0	43.6	13.3	34.1	−78.4, −47.0	0.0001
CEF13. Percentage of patients with cluster headache episode who have home oxygen therapy.	62.3	48.9	37.4	48.6	− 39.6, −10.1	0.001

## Discussion

This study’s results replayed to the primary objective (if the performance of Specialized Headache Units is better than the performance of traditional practice based on consultations). The results also provide information related to the practice of healthcare professionals treating headache and, the organization of the Healthcare system to provide adequate treatment to patients suffering headache. Indirectly, these results contribute to enhance the proposal of indicators consensual to assess the headache quality intervention.

### Performance of headaches specialized units

Comparison of indicators suggest that the performance of Specialized Headache Units was better than that of simple specialized consultations, specifically with regard to measures affecting accessibility, equity, safety and patient satisfaction. Moreover, data tend to suggest that the patient profile attended in Specialized Headache Units was more complex than that of patients attending headache consultations.

Although the patients’ improvement measured using MIDAS scale was similar between both set of patients, the trend was generally better in the Specialized Headache Units. This is because these units attend more complex conditions. Future studies could analyze what patient profile achieves greater improvement when treated in these units.

### Practice of healthcare professionals

The variability of the indicators directly associated with the practices of the professionals is lower, which indicates, on the one hand, that the same clinical references are followed in these Specialized Headache Units and, on the other, that the differences in resources have a direct repercussion in the results. However, it must be considered, as reflected in other studies [14, 15], that the level of complexity addressed, the communication mechanisms with the patient, the nursing work or the fluidity of contact with primary care professionals, modulate the differences in the indicators, although this study does not allow to discern the degree of influence of each of these factors in the results achieved.

### Organization of the Healthcare system

The data collected show that there is variability between hospitals and, therefore, in the results of the care received by patients, especially in the indicators associated with the organizational policies of the health services on which these hospitals depend (for example, accessibility to oxygen therapy or to the care of pregnant migraineurs). This result is quite similar to other studies reflecting higher variability [8, 9]. In this study Specialized Headache Units means for an effective implementation of care needed.

Overall, these results support the development of comprehensive care plans for patients with headache and are added to those other studies that advocate for the standardization of healthcare organization [16] to reduce the variability observed in the results between Specialized Headache Units. In addition to patient satisfaction, patients’ preferences [17] could be included in the future in order to choose the best treatment option for each subject.

### Headache quality indicators

The studies carried out so far with agreed indicators have focused mainly on structural and organizational issues of the healthcare process [18]. In this case, progress is made with the inclusion of outcome indicators and, although there are differences between the indicators employed, taken as a whole, they highlight that there is unnecessary variability in therapeutic management [19] and that greater organizational and clinical effort is needed to ensure an adequate level of compliance with the recommendations for offering optimal quality care [20].

Future studies should assess whether the benchmarks used to be hereafter adequate for continuous improvement.

In the absence of headache biomarkers, there is a need for a better and more objective evaluation of the quality of headache care. This study indicates that the 13 outcome indicators agreed by the headache experts [13] seem to be able to test the quality of management of patients with chronic primary headaches.

### Limitations

In each hospital there was a neurologist in charge of encoding the data, but no specific in site revision of the data was carried out. Additionally, socio-occupational factors, such as the unemployment rate, may affect any of the outcome indicators assessed, where in this study these social variables were not controlled. This study collected data during three months trying to avoid potential seasonality bias. However, future studies should consider other seasonality periods. These measures did not include infants. Indicators did not consider potential gender differences.

## Conclusions

The quality of the therapeutic approach in Specialized Headache Units has not been widely evaluated in Spain. Although there is a variability among Specialized Headache Units, the findings indicated that the performance of Specialized Headache Units was better than that of traditional specialized consultations, as well as the patient profile attended in Specialized Headache Units were more complex than the one attended in headache consultations. Future studies could analyze what profile of patients achieve better improvements being treated in Specialized Headache Units.

## Data Availability

The datasets used and/or analyzed during the current study are available from the corresponding author on reasonable request.

## Appendix I

Please rate the following statements on a scale from 0 (completely dissatisfied) to 10 (completely satisfied):

1. Easy access to the data to calculate the indicators.

**Table:**

0	1	2	3	4	5	6	7	8	9	10

2. The data sources to calculate the indicators are reliable.

**Table:**

0	1	2	3	4	5	6	7	8	9	10

3. The relevance of the indicators that have been calculated.

**Table:**

0	1	2	3	4	5	6	7	8	9	10

## Appendix II

**Table 6:** .

	Mean	Median	CV (%)
Easy access to the data to calculate the indicators	7.7	8.0	6
The data sources to calculate the indicators are reliable	8.7	9.0	6
The relevance of the indicators that have been calculated	9.5	9.5	6

*CV* coefficient of variation (standard deviation/mean)

## Abbreviations

EHF: European Headache Federation
LtB: Lifting the Burden
